# Radiation and systemic immunotherapy for metastatic uveal melanoma: a clinical retrospective review

**DOI:** 10.3389/fonc.2024.1406872

**Published:** 2024-07-04

**Authors:** Danielle H. Tran, Ryan Shanley, Alessio Giubellino, Peter H. Tang, Dara D. Koozekanani, Jianling Yuan, Kathryn Dusenbery, Evidio Domingo-Musibay

**Affiliations:** ^1^ University of Minnesota Medical School, Minneapolis, MN, United States; ^2^ Department of Medicine, University of Washington, Seattle, WA, United States; ^3^ Biostatistics Core, University of Minnesota Masonic Cancer Center, Minneapolis, MN, United States; ^4^ Department of Laboratory Medicine and Pathology, University of Minnesota, Minneapolis, MN, United States; ^5^ Department of Ophthalmology, Storm Eye Institute, Medical University of South Carolina, Charleston, SC, United States; ^6^ Department of Ophthalmology & Visual Neurosciences, University of Minnesota, Minneapolis, MN, United States; ^7^ Department of Radiation Oncology, University of Minnesota, Minneapolis, MN, United States; ^8^ Department of Medicine, Division of Hematology and Oncology, University of Minnesota, Minneapolis, MN, United States; ^9^ Department of Medical Oncology, Allina Health Cancer Institute, Minneapolis, MN, United States

**Keywords:** metastatic uveal melanoma, immunotherapy, radiation therapy (radiotherapy), retrospective study, ipilimumab, nivolumab, PD-L1

## Abstract

**Introduction:**

Metastatic uveal melanoma (mUM) is a difficult to treat disease. The liver is the primary site of metastasis in most patients, though uveal melanoma spreads widely in advanced disease. The only FDA approved immunotherapy medication for metastatic uveal melanoma is the HLA-A02:01 restricted bispecific T cell engager drug, Tebentafusp. Checkpoint inhibitor strategies and combination approaches have been tried with some limited success. We describe our experience treating patients at the University of Minnesota.

**Methods:**

Patients were included if they had biopsy-confirmed mUM. Twenty-five (25) patients meeting the criteria were identified. Medical records were reviewed and data extracted for patient baseline characteristics and response to treatments.

**Results:**

Median time to metastasis from the time of local therapy to the eye was 14.2 months (IQR; 9.3-22.0), and first site of metastasis was liver in 92% of patients. Two patients (8%) did not receive systemic therapy or radiation therapy for metastatic disease. Twenty-three (92%) patients received systemic therapy, 13 patients (52%) received ipilimumab-nivolumab as the first-line, while 4 patients (16%) received pembrolizumab. Landmark survival analysis by receipt of systemic therapy and radiation therapy treatments within 6 months of biopsy confirmed diagnosis is shown. Twenty patients (80%) received systemic therapy within 6 months of mUM diagnosis. Thirteen patients (52%) received liver directed radiation therapy within 6 months of mUM diagnosis.

**Discussion:**

Within our cohort, there was no overall survival benefit for patients receiving treatment of metastatic disease within 6 months of mUM diagnosis, versus those electing later or no treatment at all. There was remarkable clinical activity of ipilimumab and nivolumab in a subset of patients with mUM, in agreement with prior studies, and metastatic PD-L1 positive tumors were associated with a prolonged survival.

## Introduction

Uveal melanoma (UM) is the most common primary intraocular tumor found in adults and arises within the uveal tract of the eye (1). Melanocytes within the iris, ciliary body, and choroid can give rise to malignancy with a propensity for invasion and metastasis. Primary UM can be treated with enucleation or with various strategies involving radiotherapy, including plaque brachytherapy, gamma-knife stereotactic radiosurgery (GK-SRS), and proton beam therapy. While adjuvant therapy for high-risk UM has been tried with some additional benefit (2), early metastasis is common, and overall survival remains poor (3). Nearly half of UM ultimately metastasize, with the most common extraocular sites being the liver (95%), lungs (24%), bone (16%), and skin and soft tissue (11%) (4).

Due to the high rate of liver metastasis, targeted therapy to this organ is common. The selection of optimal therapeutic modality generally depends on the location and extent of metastasis. Liver-directed therapies include image-guided ablation (thermal ablation, radiofrequency ablation, or cryoablation), radioembolization [also known as selective internal radiation therapy (SIRT)], immunoembolization (IE), and transarterial chemoembolization (TACE). Benefits have also been seen with isolated hepatic perfusion and hepatic artery infusion (5). In select cases of oligometastatic disease, resection of metastatic nodules and/or stereotactic radiation therapy (SBRT) can also be considered.

The use of systemic chemotherapy (dacarbazine, fotemustine, temozolomide, cisplatin, or a combination thereof) for metastatic disease has yielded disappointing results (6). Response rates to chemotherapy were generally less than 10%, and neither single nor multiagent chemotherapy extended overall survival (OS) in patients with metastatic disease. In the last decade, immunotherapy using immune checkpoint blockade with inhibitors for programmed death 1 (PD-1) and cytotoxic T-lymphocyte-associated antigen (CTLA-4) has shown promise. While efficacy of checkpoint inhibition has radically changed the approach to treatment of cutaneous melanoma and many other solid tumors, its efficacy in metastatic UM has been less dramatic.

In a retrospective analysis, 56 patients with metastatic UM refractory to prior lines of therapy were treated with anti-PD1 or anti-PDL1 therapy. Thirty-eight patients (68%) received pembrolizumab, 16 (29%) received nivolumab, and 2 (4%) received atezolizumab. Objective responses were seen in two patients for an overall response rate of 3.6% (7). In an analysis across 14 academic medical centers, the combination of nivolumab with ipilimumab yielded an overall response rate of 11.6%, median OS of 15 months, and median progression-free survival (PFS) of 2.7 months. Overall efficacy remains considerably lower than that seen for metastatic cutaneous melanoma (8). Similarly, in a recent prospective phase II study, patients received ipilimumab 3 mg/kg with nivolumab 1 mg/kg for four cycles, followed by nivolumab maintenance therapy for up to 2 years. The primary outcome of the study was overall response rate as determined by the Response Evaluation Criteria in Solid Tumors (RECIST) 1.1 criteria. Of 33 patients evaluated for efficacy, the overall response rate was 18%, including one confirmed complete response and five confirmed partial responses (9).

More recently, tebentafusp, a bispecific T-cell engager that redirects T cells to target glycoprotein 100-positive melanoma cells, has been approved for use in metastatic UM. Tebentafusp has been shown to produce longer OS than control therapy (either single-agent pembrolizumab, ipilimumab, or dacarbazine) with OS at 1 year of 73% in the tebentafusp group and 59% in the control group (10). Notably, the treatment is limited to patients that are HLA-A*02:01 positive and requires weekly IV infusions and initial blood pressure support and monitoring. There remains a critical unmet need for additional efficacious therapies in this patient population.

Here, we describe our recent experience in treating patients with metastatic UM at the University of Minnesota. Patients during this time period received several different treatments, including SBRT, Y90 radioembolization, chemotherapy, high-dose IL-2, dual- or single-agent checkpoint inhibitors, and cellular therapy. We report clinical data, response to radiation and systemic treatments, survival, and treatment-related adverse events in evaluable subjects.

## Materials and methods

### Patients and treatment

We conducted a single-center retrospective study of patients with metastatic UM treated at the University of Minnesota between 2016 and 2022. Patients were included if they had biopsy-confirmed metastatic UM. Twenty-five patients meeting the criteria were identified. Medical records were reviewed, and data were extracted for patient baseline characteristics and response to therapy.

### PD-L1 immunohistochemistry

Biopsied metastatic lesions were assessed for expression of PD-L1 on the surface of the tumor cells at the University of Minnesota Central Pathology Laboratory or an external laboratory by means of immunohistochemical testing of formalin-fixed, paraffin-embedded tumor specimens using a monoclonal antihuman PD-L1 antibody. PD-L1 positivity was defined as having at least 1% of tumor cells showing PD-L1 staining of any intensity on the cell surface in 100 cells being evaluated. Seven patients in our study did not have PD-L1 results available.

### Adverse events and assessments

Toxicity data were abstracted from clinical notes. Toxicity events were categorized. Imaging assessments were performed every 3 months using cross-sectional computed tomography (CT), combined positron emission tomography and CT (PET-CT), and magnetic resonance imaging (MRI) modalities. Response was evaluated by the treating oncologist and reported as the best overall response (BOR) using RECIST v1.1 criteria (11).

### Statistical analysis

Survival was estimated using the Kaplan–Meier method and groups compared by log-rank test. Log-log confidence intervals are reported. OS of metastatic UM was measured from the date of biopsy-confirmed diagnosis of metastatic disease to the date of death or last known follow-up. Time to metastasis (TTM) was calculated from the date of local therapy to the eye to the date of biopsy-confirmed diagnosis of metastatic disease.

We used landmark analysis for overall survival through receipt of systemic therapy and radiation therapy. Six months after metastatic disease diagnosis was selected as the landmark time. Patients who experienced an event or were censored before the landmark were excluded from the analysis. For the analysis of OS by PD-L1 status, the full cohort was used, and the time of origin remained as the date of metastatic disease diagnosis. Statistical calculations were performed using R software, version 4.2.

## Results

### Patient characteristics

We identified 25 patients with metastatic UM treated in our medical center with confirmed biopsy. **Table 1** summarizes the baseline characteristics of the full cohort of patients. The median age at diagnosis of metastasis was 68.9 years old. All patients were Caucasian, and 13 patients (52%) were male. Median time to metastasis was 14.2 months (IQR; 9.3–22.0), and initial organ of spread was the liver in 92% of the patients. PD-L1 status was known for 18 patients; 4 were PD-L1+ and 14 were PD-L1-. PD-L1 status was not known for seven patients.

**Table 1:** Baseline patient characteristics, n = 25.

Characteristic	N=25
Age at mUM diagnosis, n (%)	
<60 years	9 (36)
≥60 years	16 (64)
Median (IQR), years	68.9 (52.2 - 77.6)
Sex, n (%)	
Male	13 (52)
Female	12 (48)
Primary Treatment, n (%)	
Plaque brachytherapy	12 (48)
Gamma knife radiosurgery	2 (8)
Proton beam Radiotherapy	3 (12)
Enucleation	6 (24)
Orbital exenteration	2 (8)
Adjuvant therapy, n (%)	
Sunitinib	2 (8)
Nivolumab	2 (8)
Time to metastasis (TTM)	
Median, months	14.2
IQR	9.3 - 22.0
Liver involvement at mUM diagnosis	
Positive	23 (92)
Negative	2 (8)
PD-L1 expression of metastasis	
Positive	4 (16)
Negative	14 (56)
Unknown	7 (28)

mUM, metastatic uveal melanoma; IQR, interquartile range.

### Treatment

Two patients (2/25) did not receive systemic therapy or radiation therapy for metastatic disease. First-line treatment regimens are summarized in **Table 2**. Of the 23 patients that received systemic therapy, 13 patients (57%) received ipilimumab–nivolumab as a first-line, while 4 patients (17%) received pembrolizumab. Three patients received first-line sequential high dose IL-2 (6 weeks) and ipilimumab–nivolumab (13%), and 2 patients received first-line carboplatin–paclitaxel (9%). Nearly all patients (21/23, 95%) received ipilimumab and nivolumab at some point during their treatment. Of the patients receiving treatment, 13 (57%) received palliative intent radiation therapy within 6 months of metastasis diagnosis (**Table 3**).

**Table 2:** First-line metastatic disease treatment regimen.

Treatments	No (%)
Ipilimumab/Nivolumab	13 (57)
HD IL-2 plus Ipilimumab/Nivolumab^a,b^	3 (13)
Pembrolizumab	4 (17)
Haploidentical NK cells^b^	1 (4)
Carboplatin/Paclitaxel	2 (9)
Treatment within 6 months^c^	21 (91)

a sequentially administered HD IL-2 and Ipilimumab/Nivolumab.

b treatment on clinical trial protocol.

c receipt of first-line systemic therapy within 6 months of metastatic disease diagnosis.

**Table 3:** Radiation treatment for metastatic disease.

Treatments	No (%)
CNS	
GK SRS	2 (10)
Pulmonary	
SBRT	1 (5)
Liver	
SBRT	5 (25)
Y-90 Radioembolization	10 (50)
Bone	
Palliative EBRT	3(15)
RT within 6 months^a^	
Yes	13 (65)
No	7 (35)

SBRT, stereotactic body radiotherapy; EBRT, external beam radiation therapy; GK SRS, gamma knife stereotactic radiosurgery; RT, radiation therapy. ^a^receipt of radiation therapy within 6 months of metastatic disease diagnosis.

### Treatment responses

**Figure 1:**
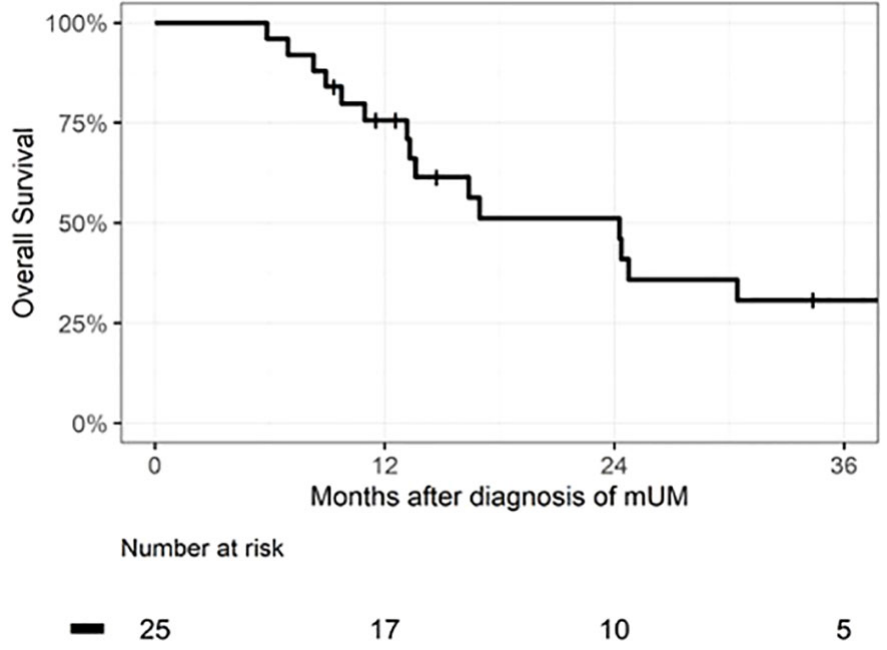
Kaplan–Meier curve of overall survival of the full cohort of patients diagnosed with metastatic uveal melanoma (mUM), n = 25 patients. Median survival time was 24 months (IQR 13–48).

**Figure 2:**
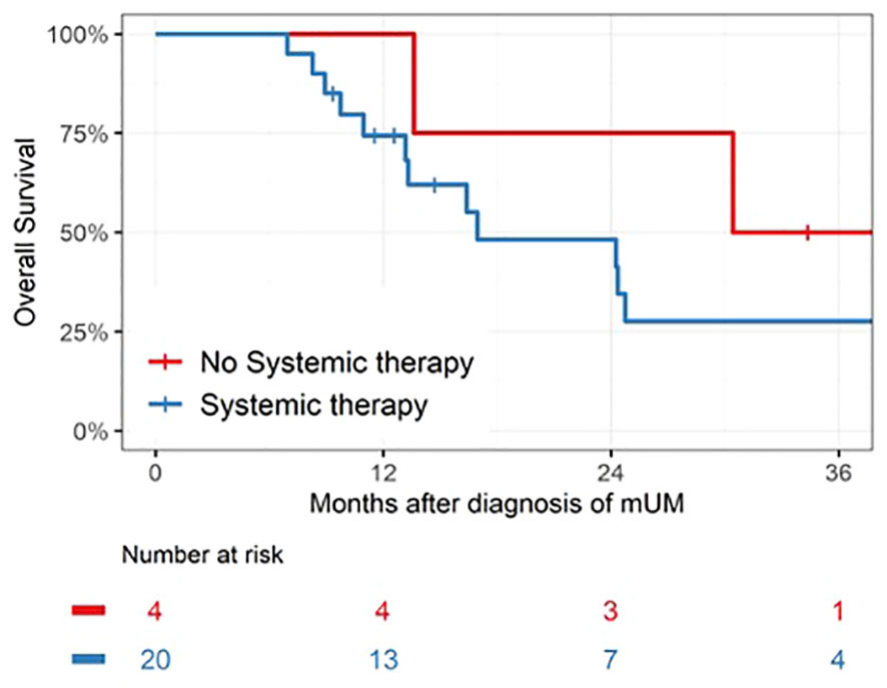
Landmark analysis of patients receiving systemic therapy within 6 months of mUM diagnosis. Median survival was 17 months (IQR 11 –50, n = 20) for the Systemic Therapy group versus 39 months (IQR 22–48, n = 4) for the No Systemic Therapy group, p = 0.57.

Median OS for the entire patient cohort was 24 months. **Figure 1** depicts the OS as measured from time of pathologic diagnosis of metastatic disease for all 25 patients. **Figure 2** depicts the landmark analysis of OS, stratified by systemic therapy treatment initiation within 6 months of metastasis diagnosis. Overall survival for the 20 treated patients was 48% at 24 months after diagnosis (95% CI: 23, 69) versus 75% (95% CI: 13, 96) for the 4 patients not treated before 6 months (p = 0.57). Two patients who began treatment after 6 months were included in the non-treated group for the landmark analysis. One patient who received systemic therapy but died within 6 months of diagnosis was not included in the landmark analysis.

**Figure 3:**
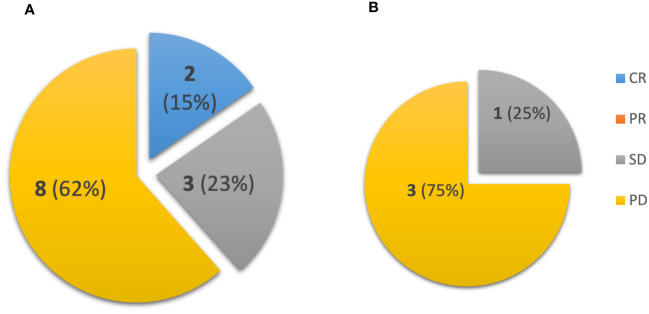
Patients were evaluated for Best Overall Response (BOR) with **(A)** first-line ipilimumab/nivolumab (n = 13), compared with **(B)** first-line pembrolizumab (n = 4). CR, complete response; PR, partial response; SD, stable disease; PD, progressive disease.

Patients receiving systemic immunotherapy were also included in the best overall response analysis to check-point blockade. In **Figure 3**, BOR for ipilimumab/nivolumab as first-line treatment was 15% versus 0% for pembrolizumab. There were two complete responses (CR) and three stable diseases (SD) with the combination of ipilimumab/nivolumab, while only one patient treated with pembrolizumab maintained stable disease.

A total of 20 patients received radiation therapy for disease palliation, described in **Table 3**. Fourteen patients received liver-directed therapy (either SBRT or Y-90 radioemoblization), with one patient initially receiving SBRT to the liver followed by Y90 radioembolization at a later time. Two patients received GK SRS for brain metastases, three patients received palliative dose radiation therapy for bone metastases, and one patient received SBRT for lung metastasis.

**Figure 4:**
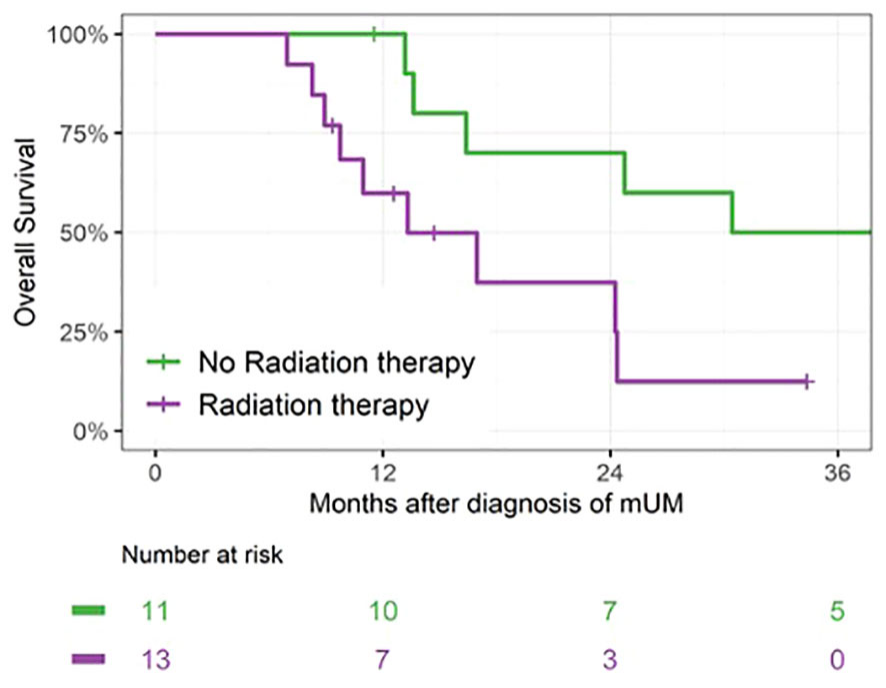
Landmark analysis of patients receiving radiation therapy within 6 months of mUM diagnosis. For patients in the Radiation Therapy group, median survival was 13 months (IQR 10–24, n = 13) versus 39 months (IQR 16–56, n = 11) for patients in the No Radiation Therapy group, p = 0.02.

A total of 13 patients received radiation therapy for metastases within 6 months of metastasis diagnosis: four received SBRT to dominant hepatic masses, eight received Y90-radioembolization to the liver (generally as two separate treatments), and one received palliative radiation to a painful bony vertebral metastasis. For the purpose of the landmark analysis, patients who initiated radiotherapy within 6 months were included in the Radiation Therapy group (n = 13). Patients who did not initiate radiotherapy within 6 months (n = 11) were counted in the No Radiation Therapy group, which included six patients who later initiated radiotherapy. There was no survival benefit seen with radiotherapy initiated within 6 months of diagnosis. OS at 24 months was 37% (95% CI: 11, 65) for the Radiation Therapy group versus 70% (95% CI: 33, 89) for the No Radiation Therapy group (p = 0.02; **Figure 4**).

**Figure 5:**
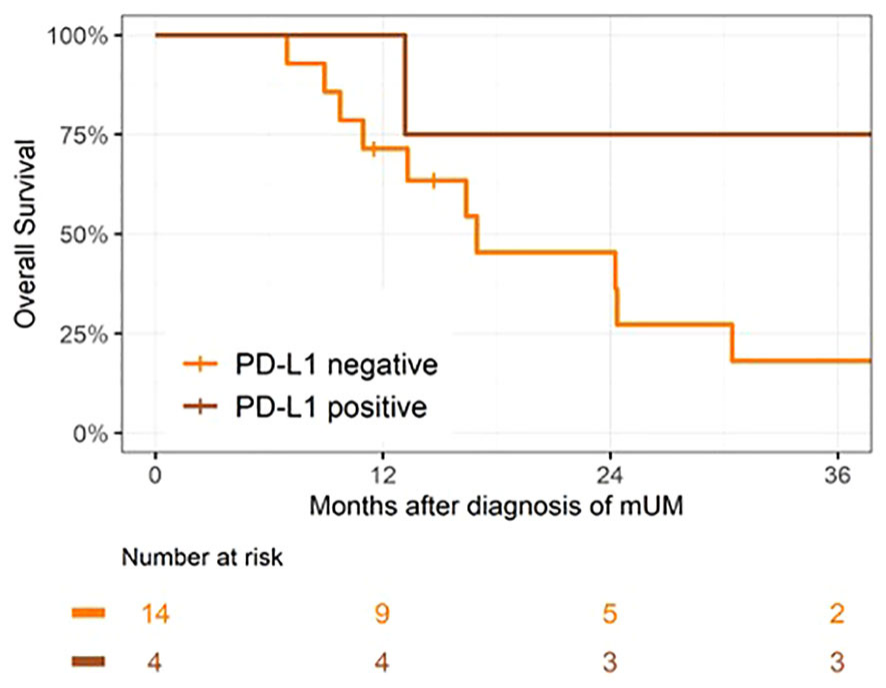
Kaplan–Meier curves of overall survival by PD-L1 status. p = 0.04.

We also assessed the potential impact of PD-L1 expression on survival. The median survival for PD- L1-negative cases (14/25, 56%) was 17 months and for PD-L1+ cases (4/25, 16%), it was 56 months (p = 0.04; **Figure 5**). All PD-L1+ patients received systemic therapy. None of the PD-L1+ received radiotherapy within 6 months.

### Immune-related adverse events

Of the 23 patients who opted to receive systemic therapy, 16 reported immune-related adverse events (irAEs) during treatment with either pembrolizumab, ipilimumab/nivolumab, or maintenance nivolumab (**Table 4**). The median number of reported irAEs were 2 per patient (range: 0–6). The severity of irAEs correlated with response. The median reported irAEs of the PD- L1-positive patients (n = 4) was 3.5 (range: 2–6), while those of the PD- L1-negative patients (n = 13) was 1 (range: 0–5), and of the unknown PD-L1 status patients (n = 3) was 2 (range: 0–2).

**Table 4:** Reported Immune-related Adverse Events (irAEs) during immunotherapy treatment, n=23.

Adverse Event (all grades)	Number of events (%)
Gastrointestinal	
Diarrhea/Colitis	8 (35)
Hepatitis	4 (17)
Pancreatitis	1 (4)
Dermatologic	
Pruritus	4 (17)
Dermatitis	3 (13)
Vitiligo	1 (4)
Hematologic	
Neutropenia	1 (4)
Endocrine	
DM type I	1 (4)
Hypothyroidism	6 (26)
Hypophysitis	3 (13)
Hypogonadism	1 (4)
Adrenal insufficiency (secondary)	2 (9)
Renal	
AKI	2(9)
Pulmonary	
Pneumonitis	3 (13)
MSK/Rheum	
Arthritis	2 (9)
Myalgia	1 (4)
Ocular	
Uveitis	1 (4)

## Discussion

In this study of 25 patients with metastatic UM, median overall survival of the entire patient cohort was 24 months. This fares well with historical accounts of median survival of 6–10 months (12, 13). Although patient numbers are small, median OS was not statistically improved in patients receiving systemic therapy within 6 months of diagnosis of metastatic disease. Similarly, no apparent survival benefit was seen with (early) radiation therapy within 6 months of diagnosis. In fact, there was a statistically significant difference in survival favoring no radiation, though later radiation therapy administration in six patients and inclusion of all four PD- L1-positive patients in the No Radiation Therapy group may have skewed results. Nonetheless, our results suggest that early systemic and radiation therapy treatments may not seemingly confer significant survival benefit for most patients. The data appear to agree with results of the meta-analysis by Rantala et al. suggesting there is no evidence for longer median OS for patients with metastatic UM by any treatment modality (3).

We did, however, note objective responses with immunotherapy and radiotherapy treatments. Systemic immunotherapy responses were highest with ipilimumab/nivolumab. BOR to ipilimumab/nivolumab as a first-line treatment was 15% versus 0% for pembrolizumab. The BOR to ipilimumab/nivolumab, irrespective of treatment line, was 24%. Our analysis showed a statistically significant difference in survival when patients were stratified by PD-L1 status. While only a small number of patients expressed PD-L1 on their tumors, survival appears improved in this small group of patients treated with checkpoint inhibition.

PD-L1 expression on metastatic UM biopsies was associated with a higher likelihood of complete responses to first-line ipilimumab/nivolumab supporting the notion that PD-L1 expression may help predict response to dual checkpoint blockade. Of the four PD- L1-positive patients in this study, there were two CRs, one PR, and one PD with ipilimumab/nivolumab. On the other hand, in the PD-L1-negative majority of patients, there were no CRs and only two PRs seen. The PD- L1-positive responses are consistent with our current understanding that high PD-L1 expression in the tumor cell population correlates positively with response to anti-PD-1 antibodies (14, 15). Unfortunately, only about 10% of primary UM tumors and just 5% of metastatic UM cells at distant sites express PD-L1 in the microenvironment (16).

PD1 checkpoint blockade in metastatic UM is less efficacious than in cutaneous melanoma likely due to differences in biology, including differences in PD-L1 and immune checkpoint molecule expression, and low mutational burden compared to cutaneous melanoma (14). However, patients with low or negative PD-L1 expression may still respond to immunotherapy, and treatment based solely on PD-L1 expression may exclude potential patient responders. For example, there is a reported case of a complete response after treatment with pembrolizumab in a patient with UM with metastases to the liver, lung, and bones (17). Another metastatic UM patient received ipilimumab plus pembrolizumab and maintained stable disease for 10 months and experienced a prolonged survival for 2 years, twice the median survival length (18). Instructively, the tumors of these two patients had a high mutational burden, and both harbored germline mutations of methyl-CpG-binding domain protein (MBD4) found in approximately 2% of UM patients (17, 18). Their exceptional response may be explained by prior studies showing that immunotherapy may be more effective in tumors with a high mutational burden and that these tumors are more likely to be recognized by CTLs due to a higher expression of recognizable neoantigens (14, 19, 20). However, UM is a tumor with a relatively low mutational burden, with an average of 0.5 per Mb sequence (21). Therefore, the probability of recognizable neoantigens is generally low in UM, likely contributing to the disappointing overall response to checkpoint inhibition compared with UV radiation-associated cutaneous melanomas. Intriguingly, one of our best overall responders had a low tumor mutational burden (TMB) of 2 Muts/Mb.

We also note that one patient initially negative for PD-L1 expression, after two cycles of high dose IL-2, had an increase in tumor cell PD-L1 expression. More than 7 months after the diagnosis of metastasis, biopsy of the patient’s liver tumor after IL-2 therapy demonstrated PD-L1 expression >5%. She was subsequently treated with two cycles of combination ipilimumab plus nivolumab and had a partial response (PR) to treatment, though complicated by severe autoimmune colitis. The patient was treated with high-dose corticosteroids, infliximab, and mycophenolate, but ultimately transitioned to hospice and received no additional cancer-directed therapy.

Our findings also suggested higher median irAEs among PD- L1-positive patients versus PD- L1-negative patients. This is consistent with the literature that supports the notion of irAEs to reflect the response to immunotherapy. In a retrospective analysis of 148 melanoma patients treated with nivolumab monotherapy, cutaneous irAEs were associated with an improved survival (22). Another meta-analysis of 48 clinical trials investigated the incidence rates of irAEs and their correlations with objective response rate (ORR) in patients with advanced solid tumors treated with nivolumab or nivolumab plus ipilimumab (23). The authors found that the ORR of nivolumab positively correlated with the incidence rate of the skin, gastrointestinal, and endocrine irAEs, but not hepatic, pulmonary, and renal irAEs. Similarly, the ORR of nivolumab plus ipilimumab was positively correlated with the incidence rate of the skin and gastrointestinal irAEs, but not endocrine, hepatic, pulmonary, and renal irAEs (23). There were higher rates of irAEs in complete responders compared to patients who did not achieve a complete response. In our patient population, patients receiving systemic treatment (n = 20) reported a range of 1 to 6 irAEs per patient. PD- L1-positive patients (n = 4) had a higher median number of 3.5 irAEs compared to the PD- L1-negative patients (n = 13) with a median of 1 irAE per patient.

Beyond PD1, alternative checkpoints may contribute to the susceptibility of metastatic UM to immune checkpoint inhibition strategies. T-cell immunoglobulin and ITIM domain (TIGIT) inhibition is being investigated in clinical trials (24, 25). Chauviin et al. originally found an upregulation of TIGIT and a co-expression of PD-1 in patients with melanoma (26). Stalhammer et al. discovered that metastatic primary UM has a higher number of TIGIT-positive cells/mm^2^ than non-metastatic UM (27). Several monoclonal antibodies have been synthesized to target TIGIT, with clinical trials on TIGIT inhibition in several cancer types underway, but further investigation for activity against metastatic UM is needed (28, 29). On the other hand, lymphocyte activation gene 3 (LAG-3) inhibition has been focused on recently as an immune checkpoint in solid tumors, including both cutaneous and UM (30–32). Opdualag (combination of relatlimab and nivolumab) has become the first FDA-approved immunotherapy to target LAG-3, with relatlimab blocking the activity of LAG-3. The RELATIVITY-047 trial compared the combination of nivolumab and relatlimab versus nivolumab alone, and showed that inhibition of two immune checkpoints (LAG-3 and PD-1) provided greater progression-free survival compared to PD-1 inhibition alone (10.1 vs. 4.6 months) in patients with advanced cutaneous melanoma (33). Ongoing trials with Opdualag in metastatic UM are currently underway (NCT04552223 and NCT05077280).

There are key limitations of this study, which are inherent to its retrospective nature, including a small number of patients and selection bias of a single-center series. There was also variability in data reporting due to patients receiving some of their care at other institutions. It was also not possible to correlate PD-L1 expression and response to radiotherapy as there were no PD- L1-positive patients treated with radiotherapy. PD-L1-positive skewing of the No Radiation Therapy group may have contributed to a measured survival difference favoring this cohort, and larger numbers of patients balanced for PD-L1 expression are needed for further verification. Adverse events were based on patient charts and descriptions, and therefore likely underreported patient-subjective adverse events (e.g., fatigue, myalgias, pruritus). Furthermore, mild toxicities and symptoms occurring between clinical visits may not have been reported.

There is an unmet need for efficacious treatment of metastatic UM. Therapeutic options are limited, and it is crucial to understand the impact of immunotherapy in combination strategies involving checkpoint inhibitors, chemotherapy, cytokines, and radiation therapy approaches.

## Conclusion

UM is a distinct subset of melanoma with decreased response to checkpoint inhibition versus cutaneous melanoma. Due to the rarity of the diagnosis and poor outcomes, there is a need for studies aimed at understanding the response to systemic and local treatments for metastatic disease. Our study showed no significant difference in survival for patients electing early treatment of their disease. There was remarkable clinical activity of ipilimumab and nivolumab in a subset of patients with UM, in agreement with prior studies. In our cohort, there were two complete responses to ipilimumab–nivolumab. Our data suggests that PD- L1-positive tumors may respond better to checkpoint blockade, and these tumors were associated with a prolonged survival. However, larger datasets are necessary to characterize the biomarkers of response to checkpoint blockade and identify molecular signatures of responders and non-responders in UM. Our data supports anti-PD-1/anti-CTLA-4 therapy as a viable treatment approach for metastatic UM patients particularly in those with PD- L1-positive tumors.

## Data availability statement

The raw data supporting the conclusions of this article will be made available by the authors, without undue reservation.

## Ethics statement

The studies involving humans were approved by University of Minnesota Institutional Review Board. The studies were conducted in accordance with the local legislation and institutional requirements. Written informed consent for participation was not required from the participants or the participants’ legal guardians/next of kin in accordance with the national legislation and institutional requirements.

## Author contributions

DT: Writing – review & editing, Writing – original draft, Methodology, Investigation, Formal analysis, Data curation, Conceptualization. RS: Writing – review & editing, Writing – original draft, Software, Methodology, Formal analysis, Data curation. AG: Writing – review & editing, Writing – original draft, Methodology, Data curation. PT: Writing – review & editing, Writing – original draft, Formal analysis, Data curation. DK: Writing – review & editing, Writing – original draft. JY: Writing – review & editing, Writing – original draft, Data curation. KD: Writing – review & editing, Writing – original draft. ED-M: Writing – review & editing, Writing – original draft, Supervision, Software, Resources, Project administration, Methodology, Investigation, Formal analysis, Data curation, Conceptualization.
